# Robust Mutation Profiling of SARS-CoV-2 Variants from Multiple Raw Illumina Sequencing Data with Cloud Workflow

**DOI:** 10.3390/genes13040686

**Published:** 2022-04-13

**Authors:** Hendrick Gao-Min Lim, Shih-Hsin Hsiao, Yang C. Fann, Yuan-Chii Gladys Lee

**Affiliations:** 1 Graduate Institute of Biomedical Informatics, College of Medical Science and Technology, Taipei Medical University, Taipei 11031, Taiwan; hendrick.san@gmail.com; 2 Division of Pulmonary Medicine, Department of Internal Medicine, School of Medicine, College of Medicine, Taipei Medical University, Taipei 11031, Taiwan; hsiaomd@gmail.com; 3 Division of Pulmonary Medicine, Department of Internal Medicine, Taipei Medical University Hospital, Taipei 11031, Taiwan; 4 IT and Bioinformatics Program, Division of Intramural, National Institute of Neurological Disorders and Stroke, National Institutes of Health, Bethesda, MD 20892, USA; fann@ninds.nih.gov

**Keywords:** COVID-19, SARS-CoV-2, mutation, variant, lineage, Illumina sequencing, cloud workflow, Common Workflow Language, parallel computing, genomics surveillance

## Abstract

Several variants of the novel severe acute respiratory syndrome coronavirus 2 (SARS-CoV-2) are emerging all over the world. Variant surveillance from genome sequencing has become crucial to determine if mutations in these variants are rendering the virus more infectious, potent, or resistant to existing vaccines and therapeutics. Meanwhile, analyzing many raw sequencing data repeatedly with currently available code-based bioinformatics tools is tremendously challenging to be implemented in this unprecedented pandemic time due to the fact of limited experts and computational resources. Therefore, in order to hasten variant surveillance efforts, we developed an installation-free cloud workflow for robust mutation profiling of SARS-CoV-2 variants from multiple Illumina sequencing data. Herein, 55 raw sequencing data representing four early SARS-CoV-2 variants of concern (Alpha, Beta, Gamma, and Delta) from an open-access database were used to test our workflow performance. As a result, our workflow could automatically identify mutated sites of the variants along with reliable annotation of the protein-coding genes at cost-effective and timely manner for all by harnessing parallel cloud computing in one execution under resource-limitation settings. In addition, our workflow can also generate a consensus genome sequence which can be shared with others in public data repositories to support global variant surveillance efforts.

## 1. Introduction

A novel coronavirus species caused a previously unidentified human pneumonia-like disease for the first time in Wuhan, China, on 12 December 2019 [1]. Later, the World Health Organization (WHO; Geneva, Switzerland) named this disease coronavirus disease 2019 (COVID-19) and the virus that caused it severe acute respiratory syndrome coronavirus 2 (SARS-CoV-2) on 11 February 2020 [2] and declared this COVID-19 a pandemic on 11 March 2020 [3] following the rapid increase in infected case numbers outside its place of origin within a short time. The virus has spread to almost all countries globally with a total of more than 270 millions cases and over 5 millions of deaths reported in for approximately 2 years since it was first detected in late 2019 [4]. Meanwhile, due to the nature of the virus itself, as the virus replicates and produces an error, replicates contain several mutations, and so the virus has evolved resulting in several variants which may be implicated in high rates of infection among human population [5]. Since May 2021, the WHO has used a new naming system for key SARS-CoV-2 variants with letters of the Greek alphabet to label variants of concern (VOCs) or variants of interest (VOIs) for easier identification by the public [6]. These correspond to their own official scientific names in the Phylogenetic Assignment of Named Global Outbreaks (PANGO) lineage nomenclature [7]. As of December 2021, among more than a thousand variants circulating around the world [8], there are five VOCs and eight VOIs with this new naming system [9] as shown in Table 1.

Meanwhile, in order to describe variants, it is critical to identify and track mutations from genome sequencing. The global health effort is shown by deposition of the sequences of SARS-CoV-2 samples from many countries in FASTA format in public data repositories, such as the Global Initiative on Sharing All Influenza Data (GISAID) EpiCoV™ [10] and the National Center for Biotechnology Information (NCBI; Bethesda, MD, USA) GenBank [11], where they have been made freely accessible since January 2020. Most of these sequences are coming from Illumina (San Diego, CA, USA) [12], which is considered the worldwide market leader for next-generation sequencing technology.

The challenge of processing SARS-CoV-2 raw sequencing data in the FASTQ format is that it is of a greater size compared to the genome sequence in FASTA format which also persists. Recently, a web-based COVID-19 analysis platform [13] was published that can process raw sequencing data in FASTQ format. However, this platform is computationally impractical for public use when dealing with large sample numbers during a pandemic event like COVID-19, since it can only deal with a single sample in each turn by default. A scalable workflow could be a solution for processing multiple samples at the same time by utilizing parallel computation [14]. Some available workflows for analyzing SARS-CoV-2 genomes are still in limited use for non-variant detection [15] or non-Illumina sequencing data [16]. Meanwhile, analyzing sequencing data of SARS-CoV-2 also remains challenging due to an enormous shortage of experts [17]. For example, many available bioinformatics tools for analyzing the SARS-CoV-2 genome [18] are complex due to the fact of their code-based utilization which need prior programming knowledge to install and implement. Therefore, implementing friendly workflows for analyzing abundant Illumina-based FASTQ raw sequencing data for variant detection purposes has becomes an urgent need during the pandemic.

In this study, we built a cloud-powered bioinformatics workflow that was optimized for mutation profiling of SARS-CoV-2 variants to accommodate limitations of both computational and expert resources. Our installation-free workflow on a specified public cloud platform can diminish the implementation barrier in resource-poor settings to allow reliable variant identification from SARS-CoV-2 Illumina raw sequencing data in a high-throughput manner by utilizing parallel computation. Mutation profiling that consists of mutation identification and annotation is the central focus of our cloud workflow, and it is complemented by the generation of consensus sequences of the SARS-CoV-2 genome which can subsequently be deposited in public data repositories to support global health efforts.

## 2. Materials and Methods

### 2.1. Data Selection

We selected sequencing data from four early VOCs (Alpha, Beta, Gamma, and Delta), which are considered highly transmissible variants in human populations [19]. These four VOC types also accounted for ~80% of the submitted SARS-CoV-2 sequences available on GISAID EpiCoV™ as of December 31, 2021, including Pango sub-lineages for each VOC, Q.1~Q.8 (aliases of B.1.1.7.1~B.1.1.7.8) sub-lineages of Alpha, B.1.351.1~B.1.351.5 sub-lineages of Beta, P.1.1~P.1.17 (aliases of B.1.1.28.1.1~B.1.1.28.1.17) sub-lineages with their own descendants of Gamma, and AY.1~AY.133 (aliases of B.1.617.2.1~B.1.617.2.133) sub-lineages with their own descendants of Delta.

**Figure 1:**
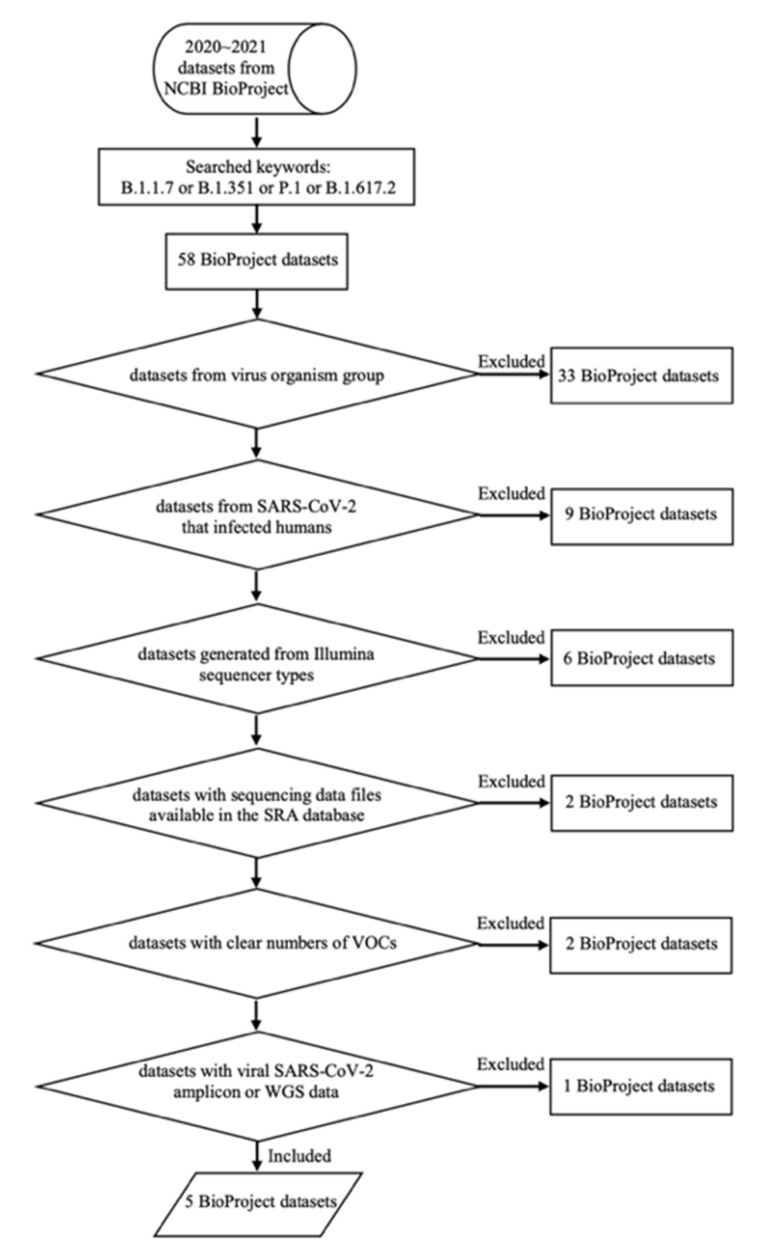
Flowchart diagram for selecting the data sets.

In this study, several data sets that collected raw sequencing data of SARS-CoV-2 from other wet-lab experiments were used to represent VOCs. The registered data sets from 2020~2021 were selected in the NCBI BioProject public repository [20] based on searches using “B.1.1.7” or “B.1.351” or “P.1” or “B.1.617.2” as keywords. The inclusion criteria were applied as follows: (1) data set from a virus organism group; (2) data sets from SARS-CoV-2 that infected humans; (3) data sets generated from Illumina sequencer types; (4) data sets with sequencing data files available in the Sequence Read Archive (SRA) database [21] at the NCBI; (5) data sets with clear numbers of VOCs; and (6) data sets with viral SARS-CoV-2 amplicon or whole-genome sequencing (WGS) data. Of 58 total BioProject data sets, 53 were ultimately excluded, leaving five data sets for further processing, which were PRJNA704235, PRJNA708134, PRJNA726840, PRJNA726871, and PRJNA733209. The flow chart diagram that shows the process of selecting these five data sets is shown in Figure 1, and a summary of each data set that accounts for a total of 55 samples subjected to more than 22 million raw sequencing reads is given in Table 2 (details of samples for each data set are available in a Supplementary Excel File, Data sets tab).

### 2.2. Data Preprocessing

We used the Cancer Genomics Cloud (CGC, https://cgc.sbgenomics.com, accessed on 13 November 2021) platform [22] from Seven Bridges Genomics (SBG; Boston, MA, USA) to further process our five selected data sets. CGC facilitates the workflow development from scratch through the Rabix Composer [23] using Common Workflow Language (CWL) [24], which is emerging as a workflow definition standard to describe analytical pipelines of bioinformatics tools for portable, scalable, and reproducible analyses. Meanwhile, the CGC also has hundreds of predefined tools or workflows which can be used for bioinformatics analytical purposes inside the platform that are publicly accessible through its web-based interface.

In the beginning, we used a predefined workflow called SRA Download and Set Metadata that implements SRA fasterq-dump (v.2.10.8) from the SRA Toolkit [25] to facilitate the automatic transfer of FASTQ raw sequencing files for all selected VOC data sets from the SRA database into the CGC platform in one execution turn. We provided the SRA metadata files in TXT format which contained the SRA accession numbers (the full list is available in a Supplementary Excel File, SRA Metadata tab) representing the FASTQ raw sequencing file for each sample of our selected VOC data sets as the input. All output read files from this workflow were then used as input for another predefined tool in the CGC called FastQC (v. 0.11.4) which implements the stand-alone FASTQC program [26] to evaluate the sequencing quality of each file. Only files that had an overall good per-base sequence quality assessment according to this tool were used for further mutation profiling purposes by our cloud workflow.

### 2.3. Mutation Profiling

#### 2.3.1. Cloud Workflow Design

**Figure 2:**
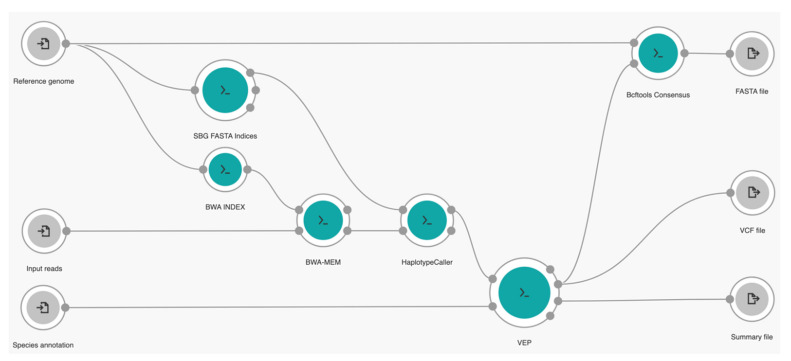
Graphical representation of our cloud workflow.

Our own tailored workflow is a series of several well-established bioinformatics tools, starting from alignment, variant calling, and variant annotation, to genome reconstruction built in the CGC platform. We used Burrows-Wheeler Alignment Maximal Exact Matches (BWA-MEM) for alignment [27], HaplotypeCaller from the Genome Analysis ToolKit (GATK) for variant calling [28], the Ensembl Variant Effect Predictor (VEP) for annotation [29], and BCFtools for consensus genome reconstruction [30]. Our workflow can run multiple times in parallel to process each sample per instance from Amazon Web Services (AWS; Amazon, Seattle, WA, USA) or the Google Cloud Platform (GCP; Google, Mountain View, CA, USA) by enabling the batch-mode option based on specified metadata criteria of input files on the platform. A layout of our cloud workflow is given in Figure 2.

In general, our workflow can be explained as follows. In the beginning, our workflow needs three different input files provided by the user: the raw sequencing read files as FASTQ, a reference genome as FASTA, and species cache information as zipped files. For this study of SARS-CoV-2 mutation profiling purposes, we used previously preprocessed files of selected VOC data sets as input reads, the original genome sequence of viral SARS-CoV-2 ribonucleic acid (RNA) with a length of 29,903 nucleotides [31] available in NCBI GenBank (NC_045512.2) as our reference genome, and annotation of a SARS-CoV-2 species cache file provided by Ensembl Genomes [32] for easier annotation purposes (available at http://ftp.ebi.ac.uk/ensemblgenomes/pub/viruses/variation/vep/sars_cov_2_vep_101_ASM985889v3.tar.gz, accessed on 13 November 2021). Then, the BWA INDEX tool was used to transform the reference genome in the full-text index in Minute space (FM-index) prior to the alignment process. At the same time, the reference genome was also processed by SBG FASTA Indices to create the FASTA dictionary and index which are later required for running GATK tools. The raw reads were aligned to the reference genome in the FM-index by BWA-MEM tool (v.0.7.17) and embedded with Biobambam2 sortmadup (v.2.0.87) [33] internally to detect and remove duplicate reads by selecting “RemoveDuplicates” for the duplicate detection option in the workflow settings. Next, the HaplotypeCaller tool (v. 4.0.2.0) was used to perform variant calling of aligned reads to generate the information of nucleic acid change positions for mutation identification purposes in the variant call format (VCF) output file [34], a standard format for recording genome polymorphism data, such as single nucleotide variations (SNVs), insertions/deletions (indels), and structural variants. Therefore, the VEP tool (v.101.0) was further used for detailed annotations of this VCF file by selecting “True” for the output sequence ontology variant class and to pick one line or block of consequence data per variant, including the transcript-specific column option, and “sars_cov_2” for the species option to generate the output of detailed annotation of VCF with its corresponding summary file. Finally, the Bcftools Consensus tool (v.1.9) was used to generate the genome sequence of raw sequencing data in FASTA format based on this VCF file and the reference genome.

#### 2.3.2. Validation

All genome sequences reconstructed by our workflow were then passed to the web-based Pango lineage assigner tool (Pangolin, https://pangolin.cog-uk.io; v.3.1.19, with Pango lineages v. accessed on 20 January 2022) [35] in a multi-FASTA way to confirm the related Pango lineage mentioned in each BioProject data set. Meanwhile, we selected the most frequent mutations, a measure determined by dividing the number of corresponding mutations of the specified variant group by the total sample available on the same variant group that was present in ≥90% of the samples in our data sets within each VOC group. The same criteria were also applied to select the most frequent mutation for each VOC in the COVID-19 CoV Genetics (CG; https://covidcg.org/?tab=report accessed on 31 December 2021) web-based tool (v. 2.4.5) [36] to validate the mutation identification results from our workflow that occurred in larger genomics population samples by directly comparing the nucleotide features from our detailed annotation of the VCF output into this COVID-19 CG web-based tool of related Pango lineages. For this validation purpose, we used COVID-19 CG data as per 31 December 2021 that considered 965,917 Alpha, 27,098 Beta, 53,556 Gamma, and 151,863 Delta samples enabled by data from GISAID EpiCoV™. Meanwhile, another web-based tool called, the University of California Santa Cruz (UCSC) SARS-CoV-2 Genome Browser [37] was used to validate annotations of key mutations found in the most frequent mutations (available at https://genome.ucsc.edu/cgi-bin/hgTracks?db=wuhCor1 accessed on 31 December 2021) based on its 2021 updated database [38].

## 3. Results

### 3.1. Cloud Workflow Performance

In our experiment, 98% of reads (22,406,134 of 22,888,595) across 110 paired-ended read files of 55 samples passed our data preprocessing step that were used as input for our defined cloud workflow in mutation profiling steps. The 98% of reads came from data sets that had no additional file when downloaded as paired ended files containing forward and reverse reads.

All data preprocessing and mutation profiling steps were performed in the CGC platform under the availability of diverse computed-optimized AWS instances. For twenty minutes, US$ 2.22, with default instance configuration settings, was spent during this study as shown in more detail in Table 3. The data preprocessing step took the most time, while our workflow that focused on mutation profiling purposes took less time due to its parallel computation capability, which works very efficiently in a timely manner to simultaneously process multiple samples in one execution turn.

### 3.2. Mutation Profiling

Our workflow successfully identified 96% of samples (53 of 55) after the lineage of constructed genome sequence output for each sample was confirmed through the web-based Pangolin tool which belonged to lineages or sub-lineages of the respective lineage mentioned in corresponding data set metadata information with two samples withdrawn: SRR13907331 and SRR13907335 from PRJNA708134 Alpha group which belonged to B.1.1.528 and B.1.1.263 lineage respectively (the constructed FASTA genome file for all 55 samples is available as a zipped file in the Supplementary Materials). Some of the samples were also confirmed to belong to the sub-lineage of the respective lineage: two samples of the Q.4 sub-lineage in the Alpha group (SRR13907332 and SRR13907333 from PRJNA708134) and one sample of the P.1.14 sub-lineage in the Gamma group (SRR14736561 from PRJNA733209). From these 96% confirmed samples, 328 mutations with SNVs dominated at 273, and the remaining 55 indels were found by taking the output of detailed annotation of the VCF file for each sample from our workflow (the summary list of mutations from these 96% confirmed samples is available in Supplementary Materials: Table S1: MutProfil “Mutation tab”, with the mutation details in the matrix format available in SNVs and Indels tab in the same file).

**Figure 3:**
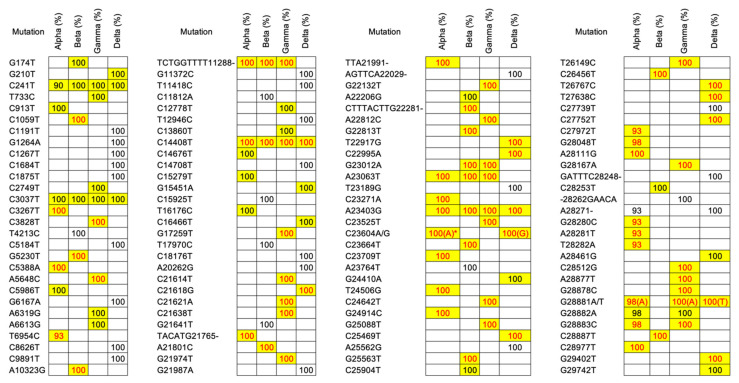
The most frequent mutations found in our variant of concern (VOC) samples. Mutation percentages highlighted in yellow denote the most common mutations listed in the COVID-19 CG for corresponding VOCs, while red mutation percentages denote key mutations listed by the UCSC SARS-CoV-2 Genome Browser. * Most mutations found in the sample, unless for its sub-lineage (Q.4) with C23604G.

We further proceeded to validate these mutations in order to observe the occurrence of common mutations in each VOC group that could generally well represent or characterize the corresponding VOCs. There were 112 most frequent mutations consisting of 104 SNVs and 8 indels (one insertion and seven deletions) across the four VOCs; 32 for Alpha, 27 for Beta, 34 for Gamma, and 42 for Delta (a special explanation for Delta must be given since it included only a single sample which was insufficient to derive the most frequent mutations; therefore, we assumed all mutations in this sample were the most frequent mutations). Our observations of these most frequent mutations also matched very well with the COVID-19 CG. Specifically, all of the most frequent mutations listed in the COVID-19 CG for each VOC (31 for Alpha, 21 for Beta, 33 for Gamma, and 20 for Delta) belonged to the list of our most frequent mutations. Meanwhile, all key mutations mentioned in the UCSC SARS-CoV-2 Genome Browser for each VOC (23 for Alpha, 15 for Beta, 24 for Gamma, and 12 for Delta) were also found in these validated most frequent mutations (see Figure 3 for a list of the most frequent and key mutations across the four VOCs). All of these key mutations occurred in the protein-coding gene region that has moderate or high mutation consequences based on detailed annotation information of the codon change from our cloud workflow output which can provide quick insights into how the mutation impacts the encoded protein (see Table 4, Table 5, Table 6 and Table 7 for more annotation details of key mutations of Alpha, Beta, Gamma, and Delta, respectively). The annotation of mutations that occurred in the open reading frame 1ab (ORF1ab) protein-coding gene from our results strongly contrasted with results in the UCSC SARS-CoV-2 Genome Browser since the annotation file did not cover all 16 non-structural proteins (nsps; nsp1~16) encoded from ORF1ab [39] due to the complexity of the annotation process in this gene region [40]. Meanwhile, results for the remaining protein-coding genes revealed a consensus with many mutations mainly found in spike (S) protein-coding genes in all VOC groups.

## 4. Discussion

We present herein a workflow that integrates several publicly available bioinformatics tools, ranging from BWA-MEM, HaplotypeCaller, and VEP to BCFtools, that can be run in a publicly available cloud platform, CGC, to process raw Illumina sequencing data in FASTQ format stored in a public SRA database from systematic open-access data sets selected in the NCBI BioProject based on some public available data for SARS-CoV-2, including the public SARS-CoV-2 reference genome in the NCBI GenBank and SARS-CoV-2 annotation data from Ensembl Genomes, for mutation profiling of SARS-CoV-2 variants. Our workflow results were highly concordant in terms of genome consensus sequence identity (to the sub-lineage level), and mutation identification and annotation when cross-validated with other dedicated SARS-CoV-2 resources, ranging from the web-based Pangolin tool to COVID-19 CG and UCSC SARS-CoV-2 Genome Browser. In addition, this study also suggested that when different data sets that come from different experiments are taken into consideration, we can still conclude the similar most frequent mutations even with limited sample sizes. Taking our experiment as an example, samples in the Alpha and Gamma groups that came from more than one data set within the sub-lineage found for each group, Q.4 for Alpha and P.1.14 for Gamma, still reflected the most frequent mutations found in larger sample sizes available from other resources. This phenomenon can be explained by the concordance of genomes deposited in the larger sample size in public repository data which were also generated from many experiments around the world resulting in high diversity levels similar to our distinct data sets used in the Alpha and Gamma groups. Meanwhile, our workflow also ensures that there is an unbiased bioinformatics analysis across data, since we used raw sequencing data as our input and applied uniform analytical tools to process these data from the beginning. This approach is critical when dealing with data that come from multiple data sets from different experiments as seen in this study due to different experiments that may have been conducted by different sequencing data analyses based on each lab’s protocol settings. Finally, our workflow that integrated many publicly available resources ensures its reproducibility for use by a wide variety of users from diverse backgrounds in their workspace by extending its capability to process their own sequencing data sets in real-world settings beyond the open-access data sets shown in this study by dragging and dropping their own sequencing files along with all necessary data using the CGC web uploader to make it run.

Understanding SARS-CoV-2 mutations is also very helpful to elucidate genomic variability that allows the virus to evade host immunity and acquire drug resistance. For example, some vaccines to prevent the COVID-19 infection were developed to target proteins of SARS-CoV-2 with the S protein as the main protein target due to its role in initiating viral entry into host cells [41]. Some mutations found in this S protein made the current vaccines less effective since they can impact antibody neutralization which makes variants within these mutations exhibit resistance to antibody-mediated immunity induced by the vaccines [42]. Meanwhile, the antiviral drug, remdesivir, is also mainly used to inhibit the SARS-CoV-2 RNA-dependent RNA polymerase (RdRp) protein (nsp12) that plays a role in viral polymerase which is essential for replicating viral RNA [43]. Any mutation to this RdRp makes the administration of remdesivir become ineffective since it affects the binding affinity of the drug [44]. Therefore, our workflow that utilizes genomics technologies is valuable for understanding the molecular underpinning of SARS-CoV-2 through profiling its mutation capability. Furthermore, the direct annotation from sequencing data within our cloud workflow can provide quick insights into where mutations exist in protein-coding genes to derive additional insights into SARS-CoV-2. Take the most frequent mutations from each VOC group in our experiment for example, there were four mutations found which overlapped in four VOCs, which were C241T, C3037T, C14408T, and A23403G. These mutations showed strong allelic associations since they were the dominant types found in the SARS-CoV-2 genome in a previous study [45]. Two non-synonymous mutations of this quartet, C14408T and A23403G, that respectively correspond to the RdRp protein P4715L and S protein D614G were found to be key mutations in this study and may impact current treatment options, while D614G was associated with an increase of human-to-human transmission efficiency [46]. They also showed significant positive correlations with fatality rates by Toyoshima et al. [47], which may explain why the disease severity increased when infected by these four VOCs [48], while the other two did not belong to the key mutation list in our experiment due to their location in the 5’ untranslated region (C241T) and having low coding consequential impacts of a synonymous mutation in the nsp3 (C3037T), which is known as the largest encoded multi-domain protein in CoV genera [49]. In addition, the raw sequencing data used as input for our cloud workflow open the possibility of precisely identifying existing or future SARS-CoV-two variants, which cannot be conducted with the current existing diagnostics test assay of reverse-transcription polymerase chain reaction (RT-PCR). Unlike sequencing, RT-PCR does not facilitate detecting changes in the SARS-CoV-2 viral genome which is key to detecting mutations in the viral variants [50].

Our workflow can be a solution to a lack of resources for analyzing the SARS-CoV-2 genome. Our workflow has an affordable cost which will be beneficial for implementation especially in those countries with limited computational and expert resources. Given the fact that many VOCs and VOIs that appeared in developing countries as determined by the United Nations [51], such as VOC Beta in South Africa, VOC Gamma and VOI Zeta in Brazil, VOC Delta and VOI Kappa in India, VOI Theta in the Philippines, VOI Lambda in Peru, and VOI Mu in Colombia, may have been circulating around the world including in those least developed countries with resource-poor settings and limited access to computational and expert resources, hence our workflow with fewer requirements is accessible online by everyone through their own local machine with internet access which can be a solution to tackle resource-limitation issues. Previously, without the workflow systems, each code-based bioinformatics tool that constructed the workflow must be installed manually in the local computer, required prior programming knowledge to perform the analysis, and works independently to analyze one sample per run [52], which all are time-consuming and not feasible especially in the time of pandemic. Even though, with workflow systems, an understanding of the syntax was still required to set up the environment first before running the entire workflow, for example, COVseq [53] and V-pipe [54] that required to install the workflow engine of Snakemake [55], resulting in an additional time that may delay mutation profiling process. Therefore, our CWL-based workflow takes advantage of particular cloud environment by packed together several pre-defined bioinformatics tools into an installation-free workflow by just copying and pasting the code in JSON format on the CGC platform. This will ensure that users with no prior programming knowledge can also directly benefit from our workflow by not necessarily deploying the analytical tools one by one anymore, since the analytical process is automatically done end-to-end along the workflow. In addition, since our workflow works through the cloud that is capable of parallel computation, the same workflow can be run in many instances to ensure robustness, while users do not necessarily have to manually perform the same analysis for each sample over and over again. Finally, our workflow can also provide great support to the global health effort of tracking the occurrence of variants around the world. Our cloud workflow is able to generate sequences of SARS-CoV-2 in FASTA format from raw sequencing data in a short time which can be deposited in public data repositories such as GISAID EpiCoV™ or NCBI GenBank. Later, these sequences can be used for any further downstream analysis, if needed, like phylogenetics analysis to track transmission, flag key mutations, or estimate reproduction numbers [56].

Our present workflow has several limitations. Although our workflow uses standardized workflow definitions in CWL, since our workflow utilizes the CGC platform-agnostic code, it might not work optimally outside the CGC platform. Therefore, prior registration in the CGC platform is needed in order to use our cloud workflow. Meanwhile, parallel computations on the CGC platform can only perform up to 80 samples in one execution turn by default. If a user wishes to use a larger number, a request should be made to the CGC team in advance. Furthermore, our workflow only works with Illumina sequencing data as the input due to genomics technological differences that exist behind each sequencing platform, while Illumina works in short read sequencing by a synthesis approach [57].

## 5. Conclusions

There are a number of SARS-CoV-2 variants circulating around the world which have made the end of the novel COVID-19 pandemic unpredictable. It is important to know particular variants based on the identified mutations of the genes, since such variants may have different virulence activities. Cloud-powered diagnostic tools have accelerated the identification of viral mutations through sequencing which has become critical to fighting COVID-19. Our workflow that collects a set of well-established bioinformatics tools for mutation profiling purposes, including viral mutation identification and annotation, can be an option to meet this demand. Our workflow designed for Illumina sequencing works in parallel, in a rapid and cost-effective manner suitable for resource-poor settings and may be best suited for application worldwide for detecting current and emerging variants so that the spread of specific variants can be limited as early as possible. In addition, our workflow is able to generate a genome sequence of SARS-CoV-2 to support global genomics surveillance by accession to public data repositories. In the future, our cloud workflow capability can be extended to predict upcoming variants with a prominent machine learning approach due to the abundant SARS-CoV-2 sequences available in public data repositories. Furthermore, our workflow can also be easily operated to detect variants of other pathogens species beyond SARS-CoV-2 as long as sequencing data, reference genome, and the related species annotation data are available.

## Figures and Tables

**Table 1:** SARS-CoV-2 variants of concern (VOCs) and variants of interest (VOIs).

Variant.	Lineage *	Alias	Place of Origin	Time of Origin	Type	Designation
α (Alpha)	B.1.1.7	-	United Kingdom	September 2020	VOC	18 December 2020
β (Beta)	B.1.351	-	South Africa	May 2020	VOC	18 December 2020
γ (Gamma)	P.1	B.1.1.28.1	Brazil	November 2020	VOC	11 January 2021
δ (Delta)	B.1.617.2	-	India	October 2020	VOC	11 May 2021
ε (Epsilon)	B.1.427/B.1.429	-	United States	March 2020	VOI	5 March 2021
ζ (Zeta)	P.2	B.1.1.28.2	Brazil	April 2020	VOI	17 March 2021
η (Eta)	B.1.525	-	multiple countries	December 2020	VOI	17 March 2021
θ (Theta)	P.3	B.1.1.28.3	Philippines	January 2021	VOI	24 March 2021
ι (Iota)	B.1.526	-	United States	November 2020	VOI	24 March 2021
κ (Kappa)	B.1.617.1	-	India	October 2020	VOI	4 April 2021
λ (Lambda)	C.37	B.1.1.1.37	Peru	December 2020	VOI	14 June 2021
μ (Mu)	B.1.621	-	Colombia	January 2021	VOI	30 August 2021
ο (Omicron)	B.1.1.529	-	multiple countries	November 2021	VOC	26 November 2021

SARS-CoV-2, severe acute respiratory syndrome coronavirus 2. * Includes all descendent lineages.

**Table 2:** Summary of selected data sets.

Data Set	No. of Samples				No. of Reads	Sequencer Type	Sequencing Strategy
	Alpha	Beta	Gamma	Delta			
PRJNA704235	-	-	3	-	1,483,054	MiniSeq	WGS
PRJNA708134	39	-	-	-	16,060,411	NovaSeq	amplicon
PRJNA726840	3	8	-	-	2,801,089	iSeq	WGS
PRJNA726871	-	-	-	1	128,048	MiSeq	amplicon
PRJNA733209	-	-	1	-	2,415,993	MiniSeq	WGS
Total	42	8	4	1	22,888,595	-	-

WGS, whole-genome sequencing.

**Table 3:** Summary of the cloud workflow performance.

Step	Name	Instance ^1^	Time ^2^	Cost ^3^
Data preprocessing	SRA Download and Set Metadata workflow	c4.8xlarge ^a^	7	0.09
	FastQC tool	c4.2xlarge ^b^	6	0.04
Mutation Profiling	Our cloud workflow *	c4.2xlarge	7	2.09
Total			20	2.22

^1^ From Amazon Web Services; ^2^ in minutes; ^3^ in US$. ^a^ Thirty-six virtual central processing units (vCPUs), 60 gigabytes (GB) of memory, and 1024 GB attached storage; ^b^ 8v CPUs, 15 GB of memory, and 1024 GB attached storage. * In parallel with one instance per sample that cost on average US $0.04 per instance.

**Table 4:** Key mutations in the Alpha VOC.

Mutation	Type	Level	Protein Coding Gene Annotation	Codon Change	Consequence for the AA Sequence	Corresponding Protein Annotation of UCSC SARS-CoV-2
C3267T	SNV	moderate	ORF1ab	aCt3002aTt	T1001I	nsp3
C5388A	SNV	moderate	ORF1ab	gCt5123gAt	A1708D	nsp3
T6954C	SNV	moderate	ORF1ab	aTa6689aCa	I2230T	nsp3
TCTGGTTTT11288-	deletion	moderate	ORF1ab	TCTGGTTTT 11023:11031-	SGF3675:3677-	nsp6
C14408T	SNV	moderate	ORF1ab	cCt14144cTt	P4715L	nsp12
TACATG21765-	deletion	moderate	S	aTACATGtc 203:208atc	IHV68:70I	S
TTA21991-	deletion	moderate	S	gtTTAt 429-431gtt	VY143:144V	S
A23063T	SNV	moderate	S	Aat1501Tat	N501Y	S
C23271A	SNV	moderate	S	gCt1709gAt	A570D	S
A23403G	SNV	moderate	S	gAt1841gGt	D614G	S
C23604A	SNV	moderate	S	cCt2042cAt	P681H	S
C23709T	SNV	moderate	S	aCa2147aTa	T716I	S
T24506G	SNV	moderate	S	Tca2944Gca	S982A	S
G24914C	SNV	moderate	S	Gac3352Cac	D1118H	S
C27972T	SNV	high	ORF8	Caa79Taa	Q27stop	ORF8
G28048T	SNV	moderate	ORF8	aGa155aTa	R52I	ORF8
A28111G	SNV	moderate	ORF8	tAc218tGc	Y73C	ORF8
G28280C	SNV	moderate	N	Gat7Cat	D3H	N
A28281T	SNV	moderate	N	gAt8gTt	D3V	N
T28282A	SNV	moderate	N	gaT9gaA	D3E	N
G28881A	SNV	moderate	N	aGg608aAg	R203K	N
G28883C	SNV	moderate	N	Gga610Cga	G204R	N
C28977T	SNV	moderate	N	tCt704tTt	S235F	N

AA, amino acid; SNV, single nucleotide variation; ORF, open reading frame; nsp, non-structural protein; S, spike; N, nucleocapsid.

**Table 5:** Key mutations in the Beta VOC.

Mutation	Type	Level	Protein Coding Gene Annotation	Codon Change	Consequence for the AA Sequence	Corresponding Protein Annotation of UCSC SARS-CoV-2
C1059T	SNV	moderate	ORF1ab	aCc794aTc	T265I	nsp2
G5230T	SNV	moderate	ORF1ab	aaG4965aaT	K1655N	nsp3
A10323G	SNV	moderate	ORF1ab	aAg10058aGg	K3353R	nsp5
TCTGGTTTT11288-	deletion	moderate	ORF1ab	TCTGGTTTT 11023:11031-	SGF3675:3677-	nsp6
C14408T	SNV	moderate	ORF1ab	cCt14144cTt	P4715L	nsp12
A21801C	SNV	moderate	S	gAt239gCt	D80A	S
CTTTACTTG22281-	deletion	moderate	S	aCTTTACTTGct719-727act	TLLA240:243T	S
G22813T	SNV	moderate	S	aaG1251aaT	K417N	S
G23012A	SNV	moderate	S	Gaa1450Aaa	E484K	S
A23063T	SNV	moderate	S	Aat1501Tat	N501Y	S
A23403G	SNV	moderate	S	gAt1841gGt	D614G	S
C23664T	SNV	moderate	S	gCa2102gTa	A701V	S
G25563T	SNV	moderate	ORF3a	caG171caT	Q57H	ORF3a
C26456T	SNV	moderate	E	cCt212cTt	P71L	E
C28887T	SNV	moderate	N	aCt614aTt	T205I	N

E, envelope.

**Table 6:** Key mutations in the Gamma VOC.

Mutation	Type	Level	Protein Coding Gene Annotation	Codon Change	Consequence for the AA Sequence	Corresponding Protein Annotation of UCSC SARS-CoV-2
C3828T	SNV	moderate	ORF1ab	tCa3563tTa	S1188L	nsp3
A5648C	SNV	moderate	ORF1ab	Aaa5383Caa	K1795Q	nsp3
TCTGGTTTT11288-	deletion	moderate	ORF1ab	TCTGGTTTT 11023:11031-	SGF3675:3677-	nsp6
C14408T	SNV	moderate	ORF1ab	cCt14144cTt	P4715L	nsp12
G17259T	SNV	moderate	ORF1ab	gaG16995gaT	E5665D	nsp13
C21614T	SNV	moderate	S	Ctt52Ttt	L18F	S
C21621A	SNV	moderate	S	aCc59aAc	T20N	S
C21638T	SNV	moderate	S	Cct76Tct	P26S	S
G21974T	SNV	moderate	S	Gat412Tat	D138Y	S
G22132T	SNV	moderate	S	agG570agT	R190S	S
A22812C	SNV	moderate	S	aAg1250aCg	K417T	S
G23012A	SNV	moderate	S	Gaa1450Aaa	E484K	S
A23063T	SNV	moderate	S	Aat1501Tat	N501Y	S
A23403G	SNV	moderate	S	gAt1841gGt	D614G	S
C23525T	SNV	moderate	S	Cat1963Tat	H655Y	S
C24642T	SNV	moderate	S	aCt3080aTt	T1027I	S
G25088T	SNV	moderate	S	Gtt3526Ttt	V1176F	S
T26149C	SNV	moderate	ORF3a	Tcc757Ccc	S253P	ORF3a
G28167A	SNV	moderate	ORF8	Gaa274Aaa	E92K	ORF8
C28512G	SNV	moderate	N	cCa239cGa	P80R	N
A28877T	SNV	moderate	N	Agt604Tgt	S202C	N
G28878C	SNV	moderate	N	aGt605aCt	S202T	N
G28881A	SNV	moderate	N	aGg608aAg	R203K	N
G28883C	SNV	moderate	N	Gga610Cga	G204R	N

**Table 7:** Key mutations in the Delta VOC.

Mutation.	Type	Level	Protein Coding Gene Annotation	Codon Change	Consequence for the AA Sequence	Corresponding Protein Annotation of UCSC SARS-CoV-2
C14408T	SNV	moderate	ORF1ab	cCt14144cTt	P4715L	nsp12
C21618G	SNV	moderate	S	aCa56aGa	T19R	S
T22917G	SNV	moderate	S	cTg1355cGg	L452R	S
C22995A	SNV	moderate	S	aCa1433aAa	T478K	S
A23403G	SNV	moderate	S	gAt1841gGt	D614G	S
C23604G	SNV	moderate	S	cCt2042cGt	P681R	S
C25469T	SNV	moderate	ORF3a	tCa77tTa	S26L	ORF3a
T26767C	SNV	moderate	M	aTc245aCc	I82T	M
T27638C	SNV	moderate	ORF7a	gTt245gCt	V82A	ORF7a
C27752T	SNV	moderate	ORF7a	aCa359aTa	T120I	ORF7a
G28881T	SNV	moderate	N	aGg608aTg	R203M	N
G29402T	SNV	moderate	N	Gat1129Tat	D377Y	N

M, membrane.

## Data Availability

The workflow source code is freely available for reproducible purposes on the Cancer Genomics Cloud platform at https://github.com/hendrick0403/MutProfil (accessed on 20 January 2022) since 2 April 2022. Meanwhile, five VOC data sets used in this study can be found in the NCBI BioProject public repository (https://www.ncbi.nlm.nih.gov/bioproject, accessed on 3 November 2021) with accession codes of PRJNA704235, PRJNA708134, PRJNA726840, PRJNA726871, and PRJNA733209.

## Supplementary Materials

The following supporting information can be downloaded at: https://www.mdpi.com/article/10.3390/genes13040686/s1, Detailed information on the data sets used in this paper and their identification performance are available in the Supplementary Materials: Table S1: MutProfil. The generated genomic sequences of all samples used in this study are also available as a zipped file: MutProfil.zip.
